# Achieving Ammonium Removal Through Anammox-Derived Feammox With Low Demand of Fe(III)

**DOI:** 10.3389/fmicb.2022.918634

**Published:** 2022-06-27

**Authors:** Lanlan Hu, Xiaohui Cheng, Guangxia Qi, Min Zheng, Yan Dang, Jiyun Li, Kangning Xu

**Affiliations:** ^1^Beijing Key Lab for Source Control Technology of Water Pollution, College of Environmental Science and Engineering, Beijing Forestry University, Beijing, China; ^2^Key Laboratory of Cleaner Production and Integrated Resource Utilization of China National Light Industry, Beijing Technology and Business University, Beijing, China; ^3^Australian Centre for Water and Environmental Biotechnology, The University of Queensland, St Lucia, QLD, Australia; ^4^School of Environment, Tsinghua University, Beijing, China

**Keywords:** Feammox, anammox, specific activity, ammonium removal, iron reduction

## Abstract

Feammox-based nitrogen removal technology can reduce energy consumption by aeration and emission of carbon dioxide. However, the huge theoretical demand for Fe(III) becomes a challenge for the further development of Feammox. This study investigated an anammox-derived Feammox process with an intermittent dosage of Fe_2_O_3_ and proposed a novel approach to reduce the Fe(III) consumption. The results showed that anammox genera *Candidatus Brocadia* and *Candidatus Kuenenia* in the seed anammox sludge significantly decreased after cultivation. The formation of N_2_ was the dominating pathway in Feammox while that of nitrite and nitrate could be neglected. Batch tests showed that specific Feammox activity of ammonium oxidation was 1.14–9.98 mg N/(g VSS·d). The maximum removal efficiency of ammonium reached 52.3% in the bioreactor with a low dosage of Fe(III) which was only 5.8% of the theoretical demand in Feammox. The removal of ammonium was mainly achieved through Feammox, while partial nitrification/anammox also played a role due to the non-power and unintentional oxygen leakage. The super-low oxygen also responded to the low demand of Fe(III) in the bioreactor because it could trigger the cycle of Fe(III)/Fe(II) by coupling Feammox and chemical oxidation of Fe(II) to Fe(III). Therefore, anammox-derived Feammox can achieve the removal of ammonium with low Fe(III) demand at super-low oxygen.

## Introduction

Anaerobic oxidation of ammonium coupling to dissimilatory iron reduction (Feammox) is part of the geochemical cycle of iron and nitrogen in the natural ecosystem, which has been widely found in wetland, freshwater, marine sediment, tropical forest soil, paddy field, etc. in recent studies (Yang et al., 2012). This biochemical process oxidizes ammonium to N_2_ (Yang et al., 2012), nitrite (Sawayama, 2006), or nitrate (Clément et al., 2005; Shrestha et al., 2009) using Fe(III) as an electron acceptor in anaerobic conditions (Equations 1–3).


(1) $$\begin{array}{l} 3\text{Fe}{\left(\text{OH}\right)}_{3}+5{\text{H}}^{+}+\text{N}{\text{H}}_{4}^{+}\to 3\text{F}{\text{e}}^{2+}+9{\text{H}}_{2}\text{O}+0.5{\text{N}}_{2},\\ {\text{Δ}}_{\text{r}}{\text{G}}_{\text{m}}=-245\text{kJ}/\text{mol} \end{array}$$

(2) $$\begin{array}{l} 6\text{Fe}{\left(\text{OH}\right)}_{3}+10{\text{H}}^{+}+\text{N}{\text{H}}_{4}^{+}\to 6\text{F}{\text{e}}^{2+}+16{\text{H}}_{2}\text{O}+\text{N}{\text{O}}_{2}^{-},\\ {\text{Δ}}_{\text{r}}{\text{G}}_{\text{m}}=-164\text{kJ}/\text{mol} \end{array}$$

(3) $$\begin{array}{l} 8\text{Fe}{\left(\text{OH}\right)}_{3}+14{\text{H}}^{+}+\text{N}{\text{H}}_{4}^{+}\to 8\text{F}{\text{e}}^{2+}+21{\text{H}}_{2}\text{O}+\text{N}{\text{O}}_{3}^{-},\\ {\text{Δ}}_{\text{r}}{\text{G}}_{\text{m}}=-207\text{kJ}/\text{mol} \end{array}$$

Theoretically, the formation of N_2_ would have a higher potential than that of nitrite and nitrate in Feammox because the Gibbs free energy of equation 1 is much lower than those of equations 2 and 3. This had been proved in experiments showing that N_2_ was the dominating product in Feammox at neutral pH while nitrite and nitrate were mainly formed in acidic conditions (Yang et al., 2012).

This geochemical phenomenon of the nitrogen cycle in nature provides a basis for the application of Feammox in the removal of nitrogen in sewage treatment. Feammox was first used as a supplementary approach to remove ammonium in some sewage treatment processes. Sawayama found that ammonium was oxidized to nitrite when ethylenediaminetetraacetic acid (EDTA)-Na-Fe(III) was dosed in the anaerobic digestion reactor of activated sludge and proposed the possibility of employing Feammox to remove aquatic nitrogen (Sawayama, 2006). Two recent studies showed that part of nitrogen was removed *via* Feammox when ferric hydroxide was used to improve the production of methane in the anaerobic digestion of activated sludge (Yang et al., 2018a; Zhu et al., 2022). In the anammox process using nitrite as the electron acceptor (equation 4), Feammox also enhanced the oxidation of ammonium as Fe(III), which could also be used as an electron acceptor (Feng et al., 2020).


(4) $$\begin{array}{l} \text{N}{\text{H}}_{4}^{+}+1.32\text{N}{\text{O}}_{2}^{-}+0.66\text{HC}{\text{O}}_{3}^{-}+0.13{\text{H}}^{+}\\ \to 0.066\text{C}{\text{H}}_{2}{\text{O}}_{0.5}{\text{N}}_{0.15}+1.02{\text{N}}_{2}+0.26\text{N}{\text{O}}_{3}^{-}+2.03{\text{H}}_{2}\text{O } \end{array}$$

It is more interesting to develop a novel treatment technology using Feammox as the dominating process for the removal of nitrogen from sewage. Compared with existing biological technologies for the removal of ammonium-based on aerobic nitrification/partial nitrification, the new technology does not need aeration, thereby reducing energy consumption and carbon emission. Inoculation of two kinds of sludge, that is, anaerobic digestion sludge and anaerobic ammonium oxidation (anammox) sludge, has been developed to trigger Feammox. The removal efficiency of nitrogen ranged from 20.1 to 82.6% in bench-scale experiments carried out under different conditions when Feammox was initiated through the inoculation of anaerobic digestion sludge (Yang et al., 2018a,b, 2019). A significant increase in chemical oxygen demand (COD) was usually observed in these studies before the start-up of Feammox due to endogenous respiration in the cultivation phase. These organic matter would inhibit the aerobic oxidation of ammonium because of the competition between organic matter and ammonium as electron donors (Jenni et al., 2014). A further study inoculated with similar seed sludge in a continuous-operating reactor showed that 53% of the influent ammonium was removed without COD while the removal efficiency decreased by 23% with COD (Zhu et al., 2022). Therefore, Feammox may be more suitable to remove ammonium from wastewater with a low C/N ratio.

Different from anaerobic sludge, anammox sludge commonly grows in a low organic environment (Jenni et al., 2014). This indicates that inoculation of anammox sludge may initiate Feammox more quickly and improve the activity of anaerobic ammonium oxidation. The removal efficiency of total nitrogen was up to 71.8% in a continuous-operating reactor employing anammox sludge as seed sludge to trigger Feammox (Li et al., 2018a). This study inferred four pathways for the anaerobic oxidation of ammonium, that is, Feammox(N_2_), Feammox(nitrite), Feammox(nitrate), and anammox. Theoretically, this corresponds to a molar ratio of Fe(III) demand to ammonium oxidized in ranges of 3–8. The high demand for Fe(III) may be a disadvantage for the Feammox technology. However, the demand for Fe(III) has not been accurately quantified and the chemical cost may be further minimized to an acceptable level because iron is the cheapest and one of the most abundant metals on the earth (US Geological Survey, 2014). Moreover, the Feammox pathways and activity still leave a lot of open questions as related publication on this novel technology is rare to the best of our knowledge. This limits the further optimization of the Fe(III) demand and the nitrogen removal from wastewater through Feammox.

Therefore, this study aims to investigate the pathway and the activity of ammonium oxidation of Feammox cultivated from anammox sludge. The cultivation was carried out in an up-flow bioreactor with continuous influent but with an intermittent dosage of Fe_2_O_3_. The transformation of nitrogen and iron and the changes in the microbial abundance were then analyzed. The Feammox pathways and activity were evaluated in batch tests inoculated with Feammox sludge obtained from the bioreactor. Several approaches were proposed to optimize the bioreactor efficiency based on the analysis of mechanisms and Feammox activity. Finally, the demand of Fe(III) for the anaerobic oxidation of ammonium was examined.

## Materials and Methods

### Materials

An up-flow reactor with a total volume of 1.2 L enclosed with a cover was used in this study (Supplementary Figure 1). Influent entered from the bottom of the reactor by a peristaltic pump (BT100-2J, Longer Pump, China), and effluent overflowed from the upper part. An S-bend pipe was equipped on the effluent pipe to avoid the transfer of oxygen into the reactor. A three-phase separator was employed in the reactor to improve the settlement of sludge back to the reaction zone (~0.76 L) and the collection of gas in the gas bag. A pH probe (E201, Leici, China) and an ORP probe (501, Leici, China) were equipped in the reaction zone. Solution and sludge in the bioreactor were agitated using a magnetic stirrer (RCT B S025, IKA, Germany).

Granular anammox sludge (Supplementary Figure 2) with typical red color was obtained from a pilot-scale anammox reactor treating wastewater with ammonium and nitrite. The reactor has been operated for more than 3 years, and the removal efficiency of nitrogen was ~98% (unpublished data).

The synthetic wastewater was composed by 100 mg N/L ammonium (NH_4_Cl), 200 mg/L MgCl_2_·6H_2_O, 136 mg/L CaCl_2_·2H_2_O, 27 mg/L KH_2_PO_4_, 500 mg/L NaHCO_3_, and 1 ml/L micronutrient solution (5,000 mg/L EDTA, 430 mg/L ZnSO_4_·H_2_O, 240 mg/L CoCl_2_·6H_2_O, 250 mg/L CuSO_4_·5H_2_O, 220 mg/L NaMoO_4_·2H_2_O, 190 mg/L NiCl_2_·6H_2_O, 210 mg/L NaSeO_4_·10H_2_O, and 14 mg/L H_3_BO_4_). A mixture gas of N_2_/CO_2_ (80%/20%) was used to remove oxygen from the synthetic wastewater for 30 min to ensure anaerobic condition (redox potential (ORP) < 0 mV) before experiments. Reagent purity Fe_2_O_3_ powder (Sinopharm Reagent Co., Ltd., China) was employed as the source of Fe(III).

### Reactor Operation

The bioreactor was operated with continuous influent and effluent at room temperature (20°C−26°C) after 200 ml of the anammox granular sludge was inoculated together with a dosage of 5 mM Fe(III) in the form of Fe_2_O_3_ powder, which had the highest ammonium removal efficiency in Feammox batch tests using different Fe sources (Zhu et al., 2022). The stirring speed was 250 rpm. Total suspended solid (TSS) and volatile suspended solid (VSS) were 2,668 mg/L and 904 mg/L, respectively, when starting the bioreactor. The hydraulic retention time was 2 d. Another 5 mM Fe(III) was supplemented on 65 d, 163 d, and 275 d, respectively, during the reactor operation. A total of 200 ml anammox granular sludge was added on 261 d. Influent and effluent were sampled every 3–6 days and filtered using 0.45 μm polyether sulfone membrane (TGMF60, Jinteng, China) before analysis. Sludge was sampled from the sludge outlet before the analysis on Feammox activity and microbiology.

### Feammox Activity Evaluation

The Feammox activity was evaluated in serum bottles in a batch test. Feammox sludge was first centrifuged at 4,000 rpm using a centrifuge (TDL-40B, Anting, China) for 10 min. Then, the supernatant was removed and the solid sludge was washed with oxygen-free de-ionized water three times to remove residual ammonium, nitrite, and nitrate. Next, the washed sludge was inoculated in a 100 ml serum bottle with 100 ml synthetic wastewater and 25 mM Fe(III) as Fe_2_O_3_ powder. The synthetic wastewater in the batch test was similar to that used in the reactor experiment but had a lower ammonium concentration of 50 mg N/L. The synthetic wastewater and the headspace in the bottle were stripped with N_2_/CO_2_ (80%/20%) for 30 min to remove oxygen. The bottle was sealed with an acrylic stopper and aluminum cover and finally tightly enclosed with tinfoil. The batch test was conducted in triplicate in an incubating shaker (THZ-98C, Yiheng, China) at 120 rpm at 32°C. The bottle was shaken artificially up and down for a complete mix before being sampled at intervals. The concentration of total Fe(II) was analyzed for the mixture of water and sludge while those of ammonium, nitrite, and nitrate were measured after filtration of the sample with 0.45 μm polyether sulfone membrane. All the following batch tests used similar experimental conditions as in this batch test except specific description.

Another similar batch test on Feammox activity was carried out using 3-time and 10-time diluted sludge obtained from the serum bottle in the first batch test. A longer cultivation time was used in this batch test in order to obtain a significant change in the concentration of ammonium with diluted sludge.

The third batch test was further conducted to investigate the effect of the initial concentration of ammonium (30 mg N/L, 90 mg N/L, and 180 mg N/L) on the Feammox activity. The seed Feammox sludge was also sampled from the bioreactor.

The effect of Fe(III) dosage on the Feammox activity was evaluated in the fourth batch test. The seed Feammox sludge was taken from the bioreactor. Fe(III) dosage of 5 mM and 25 mM was examined, while there was no dosage of Fe(III) in the blank bottles.

### Analytical Methods

Concentrations of ammonium, nitrite, and nitrate were measured according to standard methods (APHA, 2005) using an ultraviolet spectrophotometer (DR 3900, HACH, USA). Solution pH and ORP were determined by pH probe (E201, Leici, China) and ORP probe (501, Leici, China), respectively. TSS and VSS were evaluated by weight difference following standard methods (APHA, 2005). The concentration of Fe(II) in the sludge sample was analyzed following a reported method (Ding et al., 2014). In total, 1 ml of sludge was dosed with 5 ml of 0.6 M HCl solution for extraction of iron for 12 h at room temperature. Then, the mixture was centrifuged at 4,000 rpm using a centrifuge (TDL-40B, Anting, China) for 10 min. The concentration of Fe(II) in the supernatant after centrifugation was measured *via* the Ferrozine method on an ultraviolet spectrophotometer (DR 3900, HACH, USA).

Sludge was sampled on 0 d, 90 d, 150 d, and 330 d for the microbial analysis. DNA was extracted from the sludge sample using the rapid DNA soil rotation kit. Microbial abundance and community were analyzed through polymerase chain reaction (PCR) amplification of the V4-V5 region of the 16S rRNA gene using bacterial universal primers 515F (5-GTGCCAGCGCCGGG-3) and 907R (5-CCGTCAATT-CMTTTRAGTT-3) according to the method described by Li et al. (2020). The amplified products were detected using electrophoresis in 2% agarose gel. The purified PCR products were sequenced on the Illumina MiSeq platform (Meiji Biomedical Technology Co., Ltd., Shanghai, China).

Specific Feammox activity [SFA, mg N/(g VSS·d)] of ammonium oxidation was calculated as the slope of the decrease in the concentration of ammonium divided by the biomass concentration (g VSS/L) vs. time.

## Results

### Reactor Performance

#### N Removal

**Figure 1:**
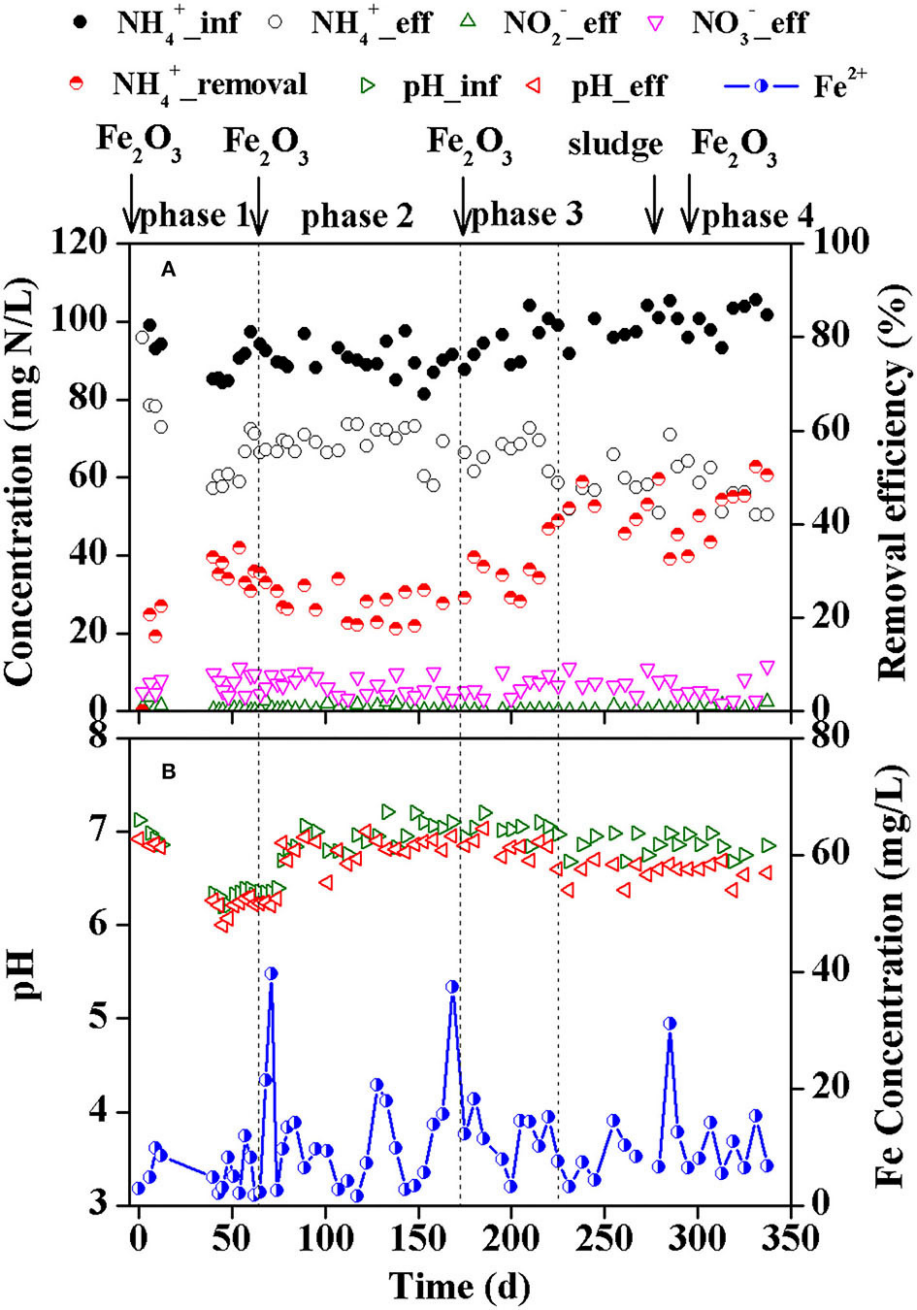
Nitrogen concentration and ammonium removal efficiency **(A)**, and pH and effluent Fe(II) concentration **(B)** during the reactor operation.

The effluent ammonium decreased gradually after starting the reactor in the first operating phase, resulting in an ammonium removal efficiency of ~32.9% on 40 d (Figure 1A). However, the removal efficiency of ammonium gradually decreased to 18.8% after 112 d in the second phase within which Feammox sludge was sampled as seed sludge for the batch tests. The third and fourth phases were carried out to improve the process efficiency. Supplementary dosage of Fe_2_O_3_ on 163 d improved the removal of ammonium, and the removal efficiency reached 40.7% on 225 d. After that, the reactor presented a relatively stable removal of ammonium defined as the fourth phase (225–337 d). On average, the removal efficiency of ammonium was 42.8% in this operating phase. However, the dosage of anammox sludge to the reactor on 261 d and a supplement of 5 mM Fe(III) on 275 d further increased the ammonium removal efficiency to 52.3% on 331 d.

The effluent nitrite was 0.05–3.60 mg N/L in the first 12 days after starting the reactor, and it was nearly stable in the following days with values lower than 1.0 mg N/L. The concentration of nitrate in the effluent was 1.8–11.5 mg N/L.

The pH in the influent was 6.20–7.21 while that in the effluent was 6.00–7.03 (Figure 1B). The average reduction in pH from influent to effluent in each sample was 0.18 with an SD value of 0.12. The concentration of total Fe(II) in the effluent was mainly in the ranges of 1.62–20.6 mg/L with serrated changes. Dosage of Fe_2_O_3_ on 65 d, 163 d, and 275 d immediately increased the concentration of total Fe(II) in the effluent to 39.7 mg/L on 71 d, 37.4 mg/L on 168 d, and 31.1 mg/L on 285 d, respectively.

#### Microbiology

**Figure 2:**
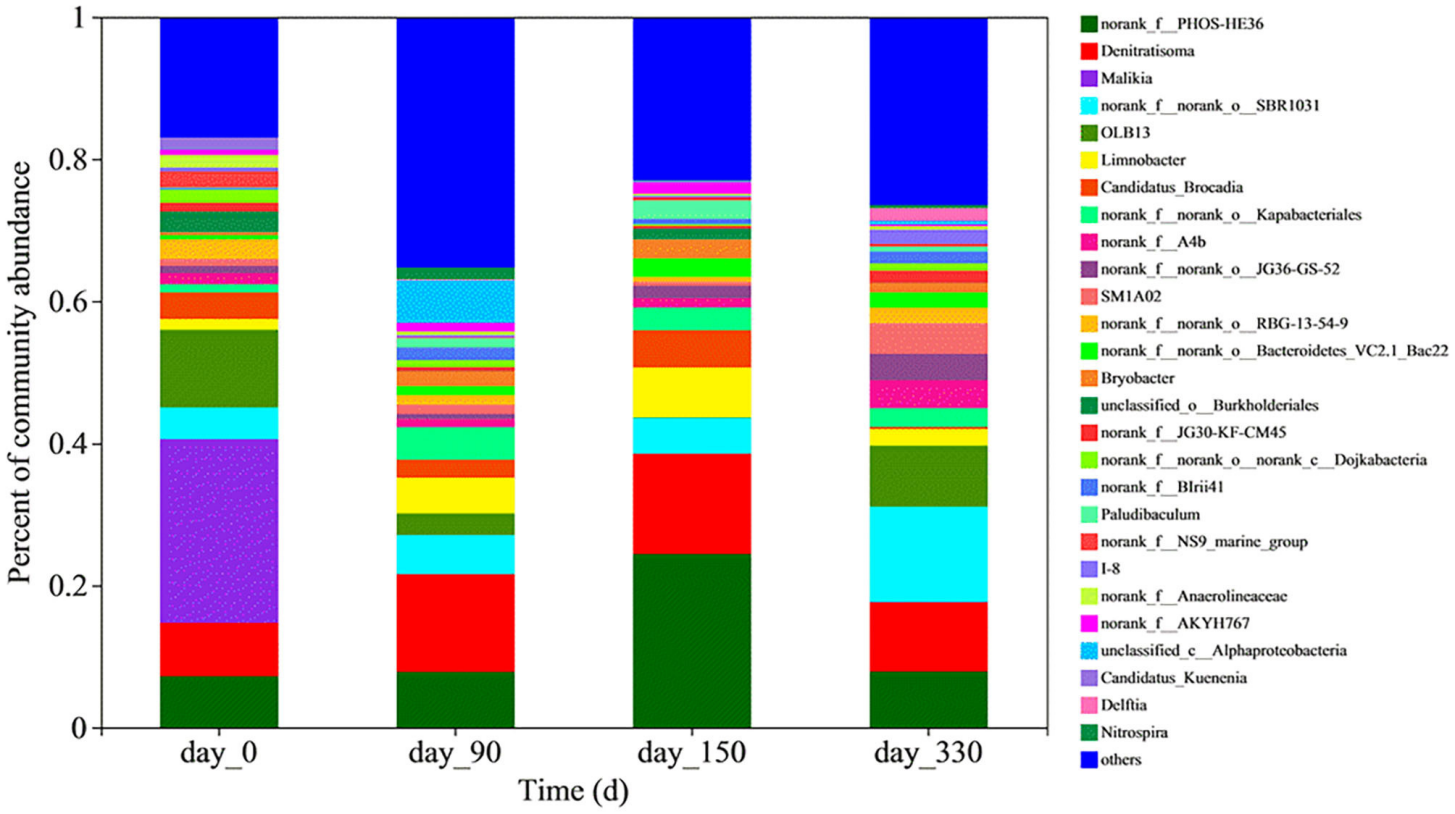
Relative abundances of bacteria at genus level on Days 0, 90, 150, and 330 in the Feammox bioreactor.

The microbial population in the bioreactor changed greatly at both the phylum level (Supplementary Figure 3) and the genus level (Figure 2). The five most abundant bacterial genera were identified to be *Malikia* (25.60%), *OLB13* (10.86%), *norank_f_PHOS-HE36* (7.13%), *Denitratisoma* (5.06%), and *norank_f_norank_o_SBR1031* (4.41%) in the seed anammox sludge. The abundances of *Malikia* and *OLB13* decreased after inoculation of the anammox sludge in the bioreactor. However, *PHOS-HE36, Denitratisoma*, and *SBR1031* were enriched after the reactor was started.

*Candidatus Brocadia* and *Candidatus Kuenenia* were the dominating anammox genera in the seed anammox sludge, with relative abundances of 3.70 and 1.62%, respectively. The abundance of *Candidatus Brocadia* genus first increased to 5.27% on 150 d and then decreased to 0.33% on 330 d. However, the *Candidatus Kuenenia* genus quickly decayed to a low relative abundance (<0.2%) after 90 d.

Nitrifying genera *Nitrosomonas* and *Nitrospira* in the seed anammox sludge comprised 0.25 and 0.0094% of the microbial population, respectively. The abundance of *Nitrosomonas* remained in ranges of 0.20–0.34% after inoculation while that of *Nitrospira* increased to 1.65% on 90 d and then decreased to 0.43% on 330 d. Some dissimilatory iron-reducing bacteria (IRB) were also enriched in the bioreactor. These bacteria whose abundances are lower than 0.50% included *Desulfovibrio, Thiobacillus, Desulfobacterota, Desulfomicrobium*, and *Geobater*.

### Feammox Activity in the Batch Tests

#### Specific Feammox Activity

**Figure 3:**
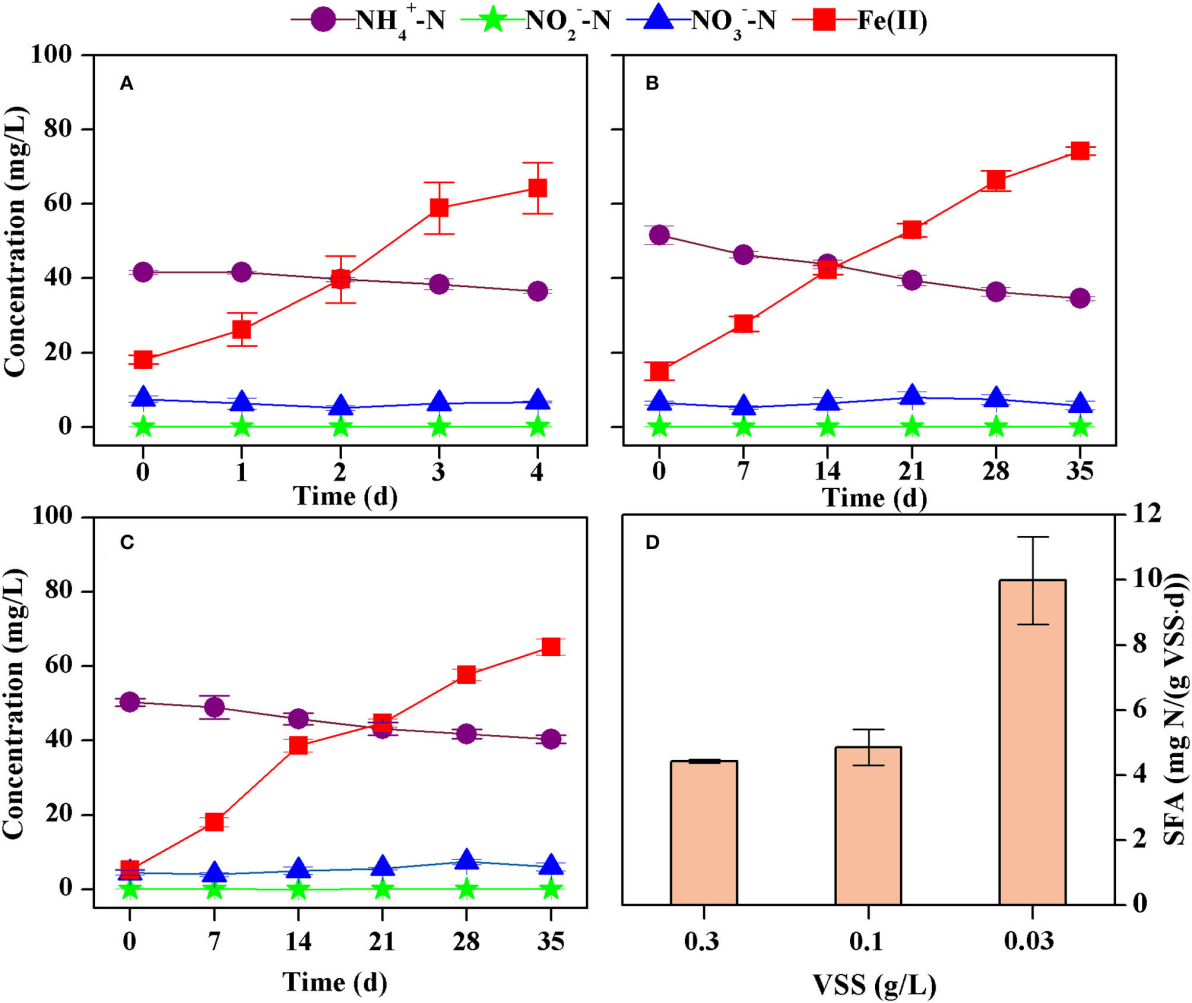
Concentrations of ammonium, nitrite, nitrate, and Fe(II) in batch tests with different VSS concentrations [**(A)** 0.30 g VSS/L, **(B)** 0.10 g VSS/L, **(C)** 0.03 g VSS/L], and the estimated specific Feammox activity of ammonium oxidation **(D)**.

The concentration of ammonium decreased from 41.5 mg N/L to 36.5 mg N/L while that of total Fe(II) increased from 18.1 mg/L to 64.2 mg/L after 4 days in the batch tests (Figure 3A). The nitrite concentration was all below 0.2 mg N/L, and the nitrate concentration ranged from 0.1 to 2.0 mg N/L. However, changes in nitrate could be neglected because analysis of variance showed there was no significant difference (*p* > 0.05) comparing the initial and the final concentrations. Thus, the formation of nitrite and nitrate could be neglected. The decrease rate of ammonium was 1.33 mg N/(L·d), estimated according to the least square method (*R*^2^ = 0.9398). This corresponds to an SFA of ammonium oxidation of 4.42 mg N/(g VSS·d) because the VSS concentration was 0.30 g/L (Figure 3D).

An almost linear decrease in ammonium and increase in Fe(II) was also observed when Feammox sludge was diluted 3 and 10 times (Figures 3B,C). The concentration of nitrite was below 0.1 mg N/L and that of nitrate was 0.1–3.5 mg N/L. There were no significant changes (*p* > 0.05) comparing their initial and final concentrations. As such, the formation of nitrite and nitrate could also be neglected. The decrease rate of ammonium was estimated to be 0.49 mg N/(L·d) (*R*^2^ = 0.9812) and 0.30 mg N/(L·d) (*R*^2^ = 0.9793) with VSS concentrations of 0.10 g/L and 0.03 g/L, respectively. This leads to SFA of ammonium oxidation of 4.85 mg N/(g VSS·d) and 9.98 mg N/(g VSS·d), respectively (Figure 3D). Therefore, a higher concentration of VSS reduced the SFA despite improving the removal of ammonium.

#### Effect of Initial Ammonium Concentration

**Figure 4:**
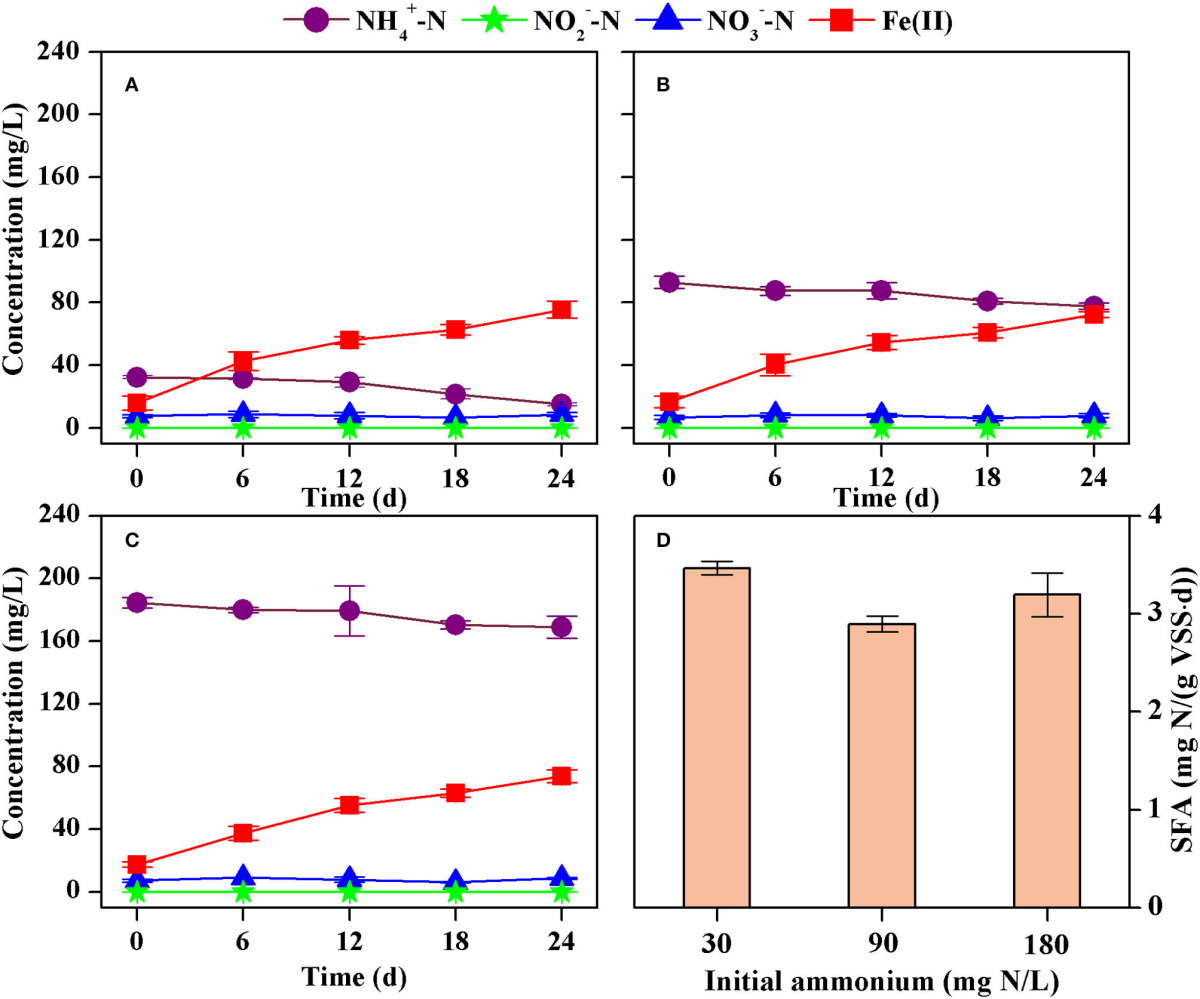
Concentrations of ammonium, nitrite, nitrate, and Fe(II) in the batch tests with different initial ammonium concentrations [**(A)** 30 mg N/L, **(B)** 90 mg N/L, **(C)** 180 mg N/L], and the estimated specific Feammox activity of ammonium oxidation **(D)**.

A similar decrease in the concentration of ammonium and an increase in the concentration of Fe(II) were obtained with different initial concentrations of ammonium (Figures 4A–C). The formation of nitrite and nitrate could also be neglected because of their irregular changes at low concentration levels. The rate of ammonium decrease was 0.74 mg N/(L·d) (*R*^2^ = 0.9024), 0.62 mg N/(L·d) (*R*^2^ = 0.9443), and 0.68 mg N/(L·d) (*R*^2^ = 0.9338) at initial ammonium of 30 mg N/L, 90 mg N/L, and 180 mg N/L, respectively. As such, the corresponding SFA of ammonium oxidation was 3.47 mg N/(g VSS·d), 2.89 mg N/(g VSS·d), and 3.19 mg N/(g VSS·d), respectively (Figure 4D). However, there were no significant differences (*p* > 0.05) in the SFAs if it was calculated for each parallel test. Thus, an increase in the initial concentration of ammonium from 30 mg N/L to 180 mg N/L did not significantly affect the SFA.

#### Effect of Fe(III) Dosage

**Figure 5:**
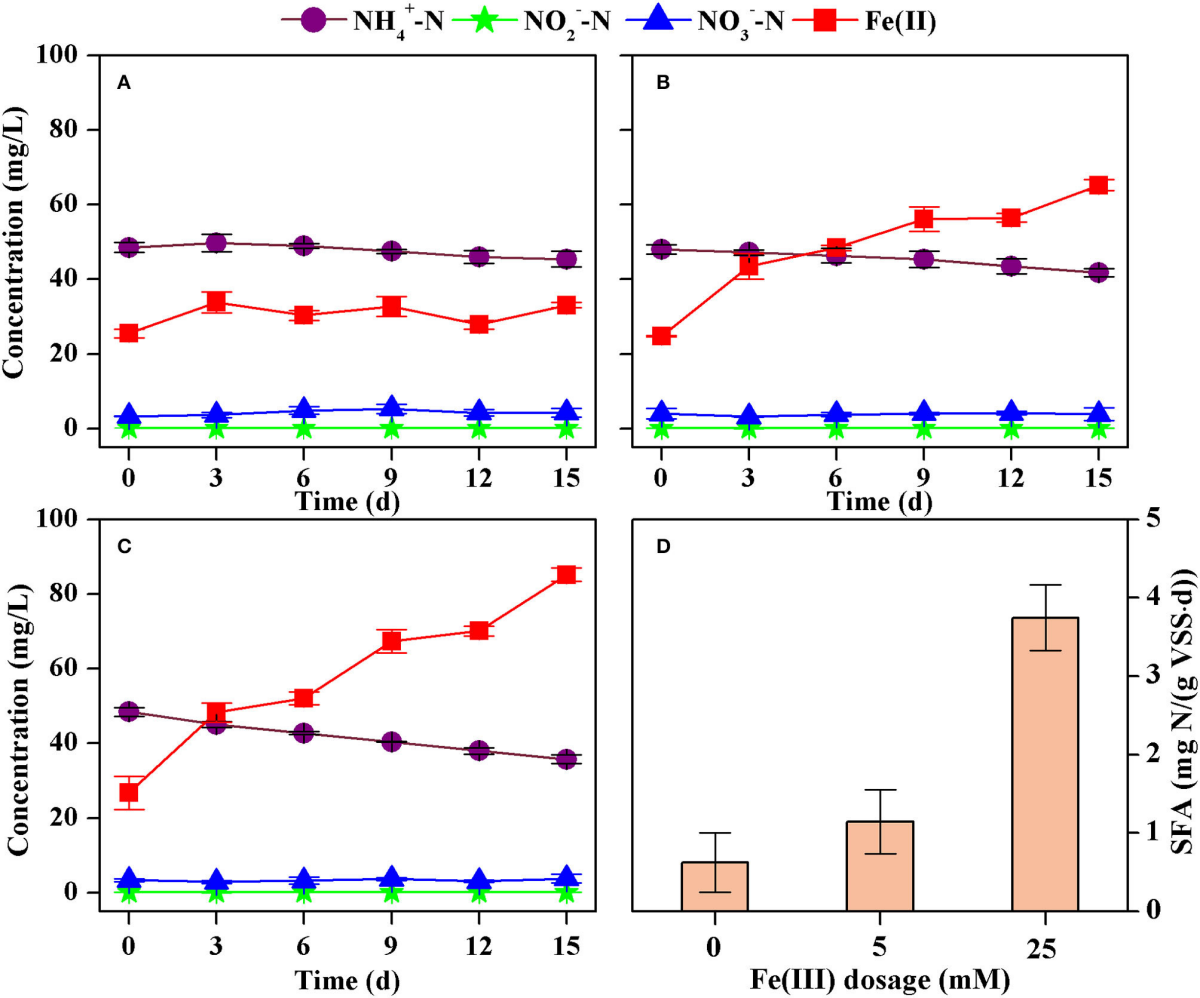
Concentrations of ammonium, nitrite, nitrate, and Fe(II) in the batch tests dosed with Fe(III) at 0 mM **(A)**, 5 mM **(B)**, 25 mM **(C)**, and the estimated specific Feammox activity of ammonium oxidation **(D)**.

**Figure 6:**
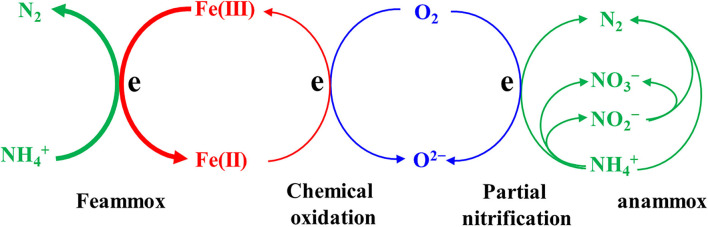
Pathways of nitrogen removal in this study.

A slight decrease in ammonium and a slight increase in Fe(II) were observed without dosage of Fe_2_O_3_ (Figure 5A), indicating the Feammox activity because sludge washing prior to the inoculation commonly removed the dissolved substances while Fe(III) contained in the sludge might remain. However, dosing 5 mM and 25 mM Fe(II) as Fe_2_O_3_ resulted in significant decrease in ammonium and increase in Fe(II) (Figures 6B,C). Again, the formation of nitrite and nitrate could be neglected in these batch tests. Calculations showed that the decrease rate of ammonium was 0.10 mg N/(L·d) (*R*^2^ = 0.8555), 0.18 mg N/(L·d) (*R*^2^ = 0.9074), and 0.60 mg N/(L·d) (*R*^2^ = 0.9538) with dosage of Fe(III) of 0 mM, 5 mM, and 25 mM, respectively. This results in an increase in the SFA from 0.62 mg N/(g VSS·d) to 3.74 mg N/(g VSS·d) when the dosage of Fe(III) increased from 0 mM to 25 mM (Figure 5D).

## Discussion

### Nitrogen Removal in the Bioreactor

The removal of ammonium and the formation of Fe(II) were observed in the bioreactor. Thus, Feammox was successfully initiated. Effluent pH was lower than influent pH, meaning a hydrion-producing Feammox process. This is consistent with the findings in an anammox-derived Feammox (Li et al., 2018a) and an anaerobic sludge-derived Feammox (Park et al., 2009). Moreover, Fe(III) and Fe(II) commonly form stable colloidal or subcolloidal hydroxide compounds at neutral and alkaline pH (Liu and Millero, 1999). As such, the reactions in equations 1–3 may also be expressed in a new form (equations 5–7), which could better explain the hydrion-producing Feammox process.


(5) $$\begin{array}{l} 3\text{Fe}{\left(\text{OH}\right)}_{3}+\text{N}{\text{H}}_{4}^{+}\to 3\text{Fe}{\left(\text{OH}\right)}_{2}+{\text{H}}^{+}+4{\text{H}}_{2}\text{O}+0.5{\text{N}}_{2}\; \end{array}$$

(6) $$\begin{array}{l} 6\text{Fe}{\left(\text{OH}\right)}_{3}+\text{N}{\text{H}}_{4}^{+}\to 6\text{Fe}{\left(\text{OH}\right)}_{2}+2{\text{H}}^{+}+4{\text{H}}_{2}\text{O}+\text{N}{\text{O}}_{2}\; \end{array}$$

(7) $$\begin{array}{l} 8\text{Fe}{\left(\text{OH}\right)}_{3}+\text{N}{\text{H}}_{4}^{+}\to 8\text{Fe}{\left(\text{OH}\right)}_{2}+2{\text{H}}^{+}+5{\text{H}}_{2}\text{O}+\text{N}{\text{O}}_{3}^{-}\; \end{array}$$

The removal efficiency of ammonium increased in the first operation phase after sludge inoculation. However, it decreased for nearly 100 days in the second phase despite 5 mM Fe(III) being supplemented to the bioreactor on 65 d. This should be caused by the sludge sampling during these days to obtain seed sludge for inoculation in the batch tests. A supplementary dosage of 5 mM Fe(III) to the bioreactor on 163 d and 275 d could improve the SFA in the bioreactor. Thus, a higher concentration of Fe(III) benefitted the process efficiency. Moreover, a supplement of new seed anammox sludge on 261 d would also favor the enrichment of Feammox bacteria, thereby increasing the ammonium removal efficiency.

The maximum efficiency of ammonium removal was 52.3% in this study, which is lower than that (~80%) obtained in a similar study dosing dissolvable FeCl_3_ together with influent in a continuous mode (Li et al., 2018a). These two studies used different Fe(III) sources. Moreover, the bioreactor was operated at room temperature in this study, while the reactor in the reference ran at 32°C. These reasons might respond to the low process efficiency in this study.

### Feammox Activity and Its Influencing Factors

The SFAs of ammonium oxidation were estimated to be 1.14–9.98 mg N/(g VSS·d) in the batch tests. Dilution of the sludge reduced the biomass of functional bacteria as measured in the form of VSS. However, the initial concentrations of ammonium and Fe(III) were the same in these three batch tests. As such, the reaction substrates were relatively more abundant for per unit of Feammox bacteria for diluted sludge. Specific anammox activity commonly positively correlates to their reaction substrates (Schielke-Jenni et al., 2015). Therefore, higher activity was obtained when the sludge was diluted. Furthermore, the removal rate of ammonium was a product of SFA and sludge VSS. The SFA was 4.42 mg N/(g VSS·d) with a VSS concentration of 0.30 g/L, while it was 9.98 mg N/(g VSS·d) with 10-time diluted sludge. Thus, more sludge obtained higher removal efficiency of ammonium despite presenting a lower SFA.

Fe(III) rather than ammonium was a limiting nutrient for Feammox bacteria in the batch tests. The Feammox sludge did not show significantly different activities of ammonium oxidation although the initial concentration of ammonium increased from 30 to 180 mg N/L. This suggests that such a concentration of ammonium was sufficient for the microbial metabolism of the Feammox bacteria in these batch tests. However, more dosage of Fe(III) obviously improved the SFA. Thus, Fe(III) with a concentration under 25 mM should be a limiting nutrient for Feammox bacteria. This is consistent with the finding that the removal of ammonium was enhanced when more Fe(III) was dosed in the bioreactor.

### Pathways of Nitrogen Removal

*Malikia* (Kutvonen et al., 2015), *PHOS-HE36* (Koenig et al., 2005), and *Denitratisoma* (de Almeida Fernandes et al., 2018) genera were all reported as heterotrophic denitrifiers. There were nearly no organics in the influent and the effluent nitrate ranged from 1.8–11.5 mg N/L in this study. This indicates that denitrification should be limited to a low level in the bioreactor. It was reported that heterotrophic bacteria, such as *OLB13* and *SBR1031*, could benefit the stability of the anammox microbial system (Su et al., 2022). Furthermore, *Denitratisoma* was found to coexist with anammox genera in anammox sludge (de Almeida Fernandes et al., 2018; Su et al., 2022). Thus, *PHOS-HE36* and *Denitratisoma* might also play a similar role as *OLB13* and *SBR1031* in the Feammox bioreactor.

At present, there is little knowledge about the functional bacteria in Feammox. A strain *Acidimicrobiaceae* sp. A6 isolated from riparian wetland soils was the only reported ammonium-oxidizing iron reducer (Huang and Jaff, 2018). Feammox was further achieved in an A6-augmented constructed wetland and A6-inoculated microbial electrolysis cells (Ruiz-Urigüen et al., 2018, 2019; Shuai and Jaff, 2019). However, A6 was not detected in the Feammox sludge obtained in this study. Most of the other studies reported that Feammox was positively related to the enrichment of IRB, for example, *Geobacter* (Zhou et al., 2016; Yang et al., 2018b; Ahmed et al., 2021; Zhu et al., 2021, 2022), *Geothris* (Ahmed et al., 2021), *Pseudomonas* (Li et al., 2019; Yang et al., 2019; Zhu et al., 2021), *Desulfovibrio* (Zhou et al., 2016), *Desulfosporosinus* (Yang et al., 2019), *Desulfomicrobium* (Yang et al., 2019), *Dechloromonas* (Yang et al., 2019, 2021a), and *Clostridium* (Yang et al., 2021a). Based on these previous studies and typical clarification of IRB (Lovley et al., 2004), the bioreactor in this study enriched five IRB genera including *Desulfovibrio, Thiobacillus, Desulfomicrobium, Geobater*, and *Desulfobacterota*. This is a signal for the successful initiation of the Feammox process besides the removal of ammonium and the formation of Fe(II). Relative abundances of the five IRB ranged from 0.1 to 0.5%. This is lower than the reported abundances of *Geobacter* and *Pesudomonas* in an anaerobic sludge-derived Feammox (Yang et al., 2019) and in a paddy soil-derived Feammox (Li et al., 2019), but comparable to the reported abundance of *Geobacter* in another anaerobic sludge-derived Feammox (Yang et al., 2021b). This suggests that these IRB would probably play a role in Feammox.

The dominating anammox genera *Candidatus Brocadia* and *Candidatus Kuenenia* decreased in the Feammox bioreactor. This should be caused by a lack of anammox reaction substrate, that is, nitrite whose concentration was lower than 1 mg N/L. However, *Candidatus Brocadia* still remained in a certain relative abundance. Some anammox bacteria including *Candidatus Kuenenia, Candidatus Scalindua*, and *Candidatus Brocadia* were suggested to participate in the Feammox process because they were enriched in an anammox-derived Feammox reactor (Li et al., 2018a). Nonetheless, more evidence is essential to support this inference that anammox bacteria can oxidize ammonium using Fe(III) as an electron acceptor in anaerobic conditions. The remained *Candidatus Brocadia* more likely consumed nitrite through the anammox process (equation 4) if Feammox produced nitrite (equation 2) as there was no nitrite accumulation both in the bioreactor and in the batch tests.

Interestingly, aerobic nitrifiers *Nitrosomonas* and *Nitrospira* were enriched in the bioreactor. *Nitrosomonas* is typical ammonia-oxidizing bacteria (AOB) and *Nitrospira* is a typical nitrite-oxidizing bacteria (NOB). Enrichment of *Nitrospira* was also observed in the Feammox system coupled with nitrate-dependent Fe(II) oxidizing (Li et al., 2018b). *Nitrosomonas* appeared in anaerobic-derived Feammox reactors (Zhu et al., 2021, 2022; Cao et al., 2022). AOB and NOB were also detected in an anaerobic sludge-derived Feammox (Zhu et al., 2022). An ammonia-oxidizing archaea (AOA), *Nitrososphaeraceae*, was enriched in a paddy soil-derived Feammox (Li et al., 2019). The functional bacteria oxidizing ammonia is still unclear, whereas IRB has been considered a key bacteria reducing Fe(III) to Fe(II) in Feammox. It was suggested that AOA (Li et al., 2019) and AOB (Cao et al., 2022; Zhu et al., 2022) might play a role in ammonia oxidation associated with Feammox. Nonetheless, some other studies on Feammox did not report similar results. AOB was not found through detection on nitrifying bacterial gene *amoA* in a Femmox process using nitrate as a terminal electron acceptor (Yang et al., 2020). AOA and AOB were not detected even in a Feammox process with intermittent aeration (Yang et al., 2021a). Actually, nitrifying bacteria including AOA, AOB, and NOB are commonly sensitive to oxygen (Gujer, 2010). Therefore, another explanation for the detection of nitrifiers in this study may be the leakage of oxygen into the Feammox system despite routine measures being taken to achieve anaerobic conditions.

Possible aerobic oxidation of ammonium may cover the understanding of Feammox. However, the relatively high concentration of Fe(II) and the approximately linear increase in Fe(II) suggested that the influence of oxygen could be neglected in the batch tests using sealed serum bottles because Fe(II) could be oxidized chemically and rapidly by low oxygen content while biological aerobic nitrifying was slow (Yang et al., 2021a). Thus, the Feammox activity should be credible in the batch tests. In this study, there was no significant accumulation of nitrite and nitrate in all the batch tests. One explanation is that the formation of N_2_ was the only pathway of Feammox, which is consistent with the experimental results reported in a previous study (Yang et al., 2012) and the theoretical conclusion based on thermodynamic analysis. However, the accumulation of nitrite and nitrate was still observed in some Feammox processes, indicating the formation of nitrite and nitrate *via* Feammox (Yang et al., 2018a; Zhu et al., 2022). Thus, another explanation is that Feammox also produced nitrite and nitrate besides N_2_, while nitrite was further removed through anammox (equation 4), and nitrite and nitrate were removed by NO*x*-dependent ferric oxidation (NDFO, equations 8 and 9), which was considered as a possible pathway together with Feammox (Oshiki et al., 2013; Yang et al., 2018b).


(8) $$\begin{array}{l} 6\text{F}{\text{e}}^{2+}+8{\text{H}}^{+}+2\text{N}{\text{O}}_{2}^{-}\to 6\text{F}{\text{e}}^{3+}+4{\text{H}}_{2}\text{O}+{\text{N}}_{2},\\ {\text{Δ}}_{\text{r}}{\text{G}}_{\text{m}}=-438.4\text{kJ}/\text{mol} \end{array}$$

(9) $$\begin{array}{l} 10\text{F}{\text{e}}^{2+}+12{\text{H}}^{+}+2\text{N}{\text{O}}_{3}^{-}\to 10\text{F}{\text{e}}^{3+}+6{\text{H}}_{2}\text{O}+{\text{N}}_{2},\\ {\text{Δ}}_{\text{r}}{\text{G}}_{\text{m}}=-457.2\text{kJ}/\text{mol} \end{array}$$

However, nitrate significantly formed in the bioreactor, suggesting that at least the pathway of NDFO reducing nitrate was denied. Moreover, the Gibbs free energy of equation 9 is lower than that of equation 8, meaning that nitrate-dependent ferric oxidation is prior to nitrite-dependent ferric oxidation. This indicates that both the reactions of NDFO can be neglected. Feammox(nitrate) probably did not contribute to the ammonium oxidation in the batch tests because NDFO was the only approach to remove nitrate. The Gibbs free energy of equation 3 is lower than that of equation 2, which means that nitrate has a higher formation potential than nitrite in Feammox. Accordingly, Feammox(nitrite) could be neglected in the batch tests. Therefore, the formation of N_2_ was the only pathway of Feammox in this study.

Nonetheless, nitrate is formed in the bioreactor. ORP in the bioreactor commonly ranged from −211 to 175 mV, which did not ensure a strict anaerobic environment. The removal of ammonium in the control bioreactor without Fe(III) also indicated possible oxygen leakage despite the bioreactor system being isolated from the air as far as possible. As such, leakage of oxygen to the reactor could be an explanation for the formation of nitrate in the bioreactor, particularly considering the enrichment of *Nitrosomonas* and *Nitrospira*. Nitrite is more likely to accumulate under low dissolved oxygen than nitrate (Schielke-Jenni et al., 2015). In this case, anammox would likely be initiated by the remaining *Candidatus Brocadia*. This indicates that a combination of partial nitrification and anammox (PN/A) produced nitrate (equation 4) in addition to nitrification.

In general, the pathways of nitrogen removal included the Feammox process and PN/A (Figure 6). Calculations were further carried out to quantify the possible pathway of ammonium oxidation, taking phase 4 of the bioreactor operation as an example. The average removal efficiency of ammonium was 42.8% in this phase while that was 14.4% in the control bioreactor. Thus, the contribution of Feammox to the ammonium removal was estimated to be approximately 66.4%, while aerobic nitrifying contributed about 33.6% if it was assumed that oxygen leakage was the same in the two bioreactors. Therefore, Feammox played a dominating role in the removal of ammonium, and PN/A played a secondary role.

### Demand for Fe(III) for Oxidation of Ammonium

The theoretical molar ratio of Fe(III) consumed to ammonium removed is determined by the anaerobic oxidation product of ammonium, that is, 3 for the production of N_2_, 6 for the production of nitrite, and 8 for the production of nitrate. However, the demand for Fe(III) decreases when NDFO works to trigger the cycle of Fe(III)/Fe(II) (Li et al., 2021; Yang et al., 2021a). According to the combination of equations 2 and 8 and that of equations 3 and 9, the molar ratio of Fe(III) consumed to ammonium removed still equals to 3 when the final product of Feammox is N_2_. This conforms to the principle of electron conservation between reduction of Fe(III) to Fe(II) and oxidation of N(−3) to N(0). Moreover, the formation of N_2_ through Feammox was the only pathway in the batch tests and the formations of nitrite and nitrate could be neglected. Therefore, the molar ratio of Fe(III) consumed to ammonium removed would be approximately 3 whether NDFO played a role in the batch tests.

**Figure 7:**
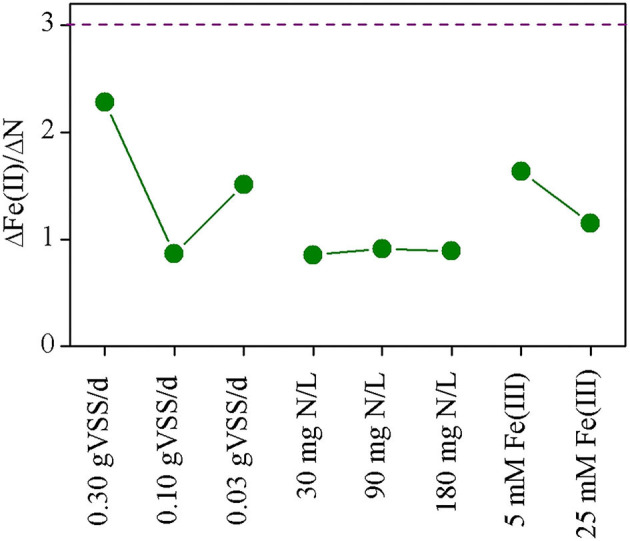
Molar ratio of Fe(II) produced to ammonium removed in the batch tests.

Fe(III) consumed was equaled to Fe(II) produced because Feammox is the only pathway in the batch tests. The molar ratio of Fe(II) produced to ammonium removed in the batch tests was estimated to be 0.87–2.28 (Figure 7), which is lower than the theoretical value. One possible reason might be the formation of insoluble ferrous salts, such as Fe_2_(PO_4_)_3_·7H_2_O [solubility product = 10^−36.00^ (Nriagu, 1972) or 10^−40.74^ (Liu et al., 2018)] and Fe_2_(CO_3_)_3_ [solubility product = 10^−10.59^ (Gustafsson, 2012)], which may settle at the bottom of serum bottles and could not be sampled prior to analysis of total Fe(II).

Different from the batch tests, the bioreactor experiment showed complicated pathways for ammonium removal. In phase 4 of bioreactor operation, the contribution of an aerobic nitrifying pathway to the removal of ammonium was estimated to be 33.6%, thereby reducing the ratio of Fe(II) produced to ammonium removed. Considering the ammonium removed in the Feammox pathway, the average molar ratio of Fe(II) produced in the effluent to ammonium removed was estimated to be 0.13 and the ratio of Fe(III) dosage to ammonium removed was 0.17, a much lower value than the value obtained in the batch tests. The molar ratio of Fe(III) dosage to ammonium removed in the bioreactor was only 5.8% of the theoretical demand *via* Feammox(N_2_). In addition, there should be other reasons for the low formation of Fe(II). Ferrous hydroxide, the main form of Fe(II) ion at neutral pH, was easily absorbed on sludge due to electrostatic forces in the wastewater treatment system (Prot et al., 2019). The up-flow bioreactor used in this study could effectively retain sludge and also ferrous hydroxide, thereby reducing the loss of Fe(II) with the effluent. Most importantly, Fe(II) retained in the reactor could be quickly oxidized due to the low dissolved oxygen because the chemical oxidation was much quicker than aerobic nitrifying (Yang et al., 2021a). Then, a combination of Feammox and chemical Fe(II) oxidation initiated the cycle of Fe(III)/Fe(II) (Yang et al., 2021a), thereby reducing the demand for Fe(III). Compared with the technology to initiate Fe(III)/Fe(II) cycle through intermittent aeration (Yang et al., 2021a), this study used non-power and unintentional oxygen leakage to effectively reduce the demand for Fe(III) for the oxidation of ammonium through Feammox.

Nonetheless, aerobic nitrifying produced nitrate in this study, which greatly reduced the removal efficiency of total nitrogen. Feammox might be inhibited because it relies on iron reduction in an anaerobic environment. Thus, the control of oxygen leakage should be addressed in further studies. Moreover, more dosage of Fe(III) may further improve the removal of nitrogen because the dosage was still comparatively low than most of the other studies (Park et al., 2009; Yang et al., 2019; Zhu et al., 2022). However, a high concentration of Fe(III) induced coagulation of activated sludge (Diamadopoulos et al., 2007). This may decrease the Feammox activity and the removal of ammonium. In addition, more dosage of Fe(III) also means high demand for chemicals, thereby increasing the process cost. Therefore, it is essential to determine a reasonable range of the demand of Fe(III) at controlled oxygen in Feammox in further studies.

## Conclusion

The SFA of ammonium oxidation was 1.14–9.98 mg N/(g VSS·d) in the batch tests. Feammox(N_2_) was the dominating pathway, while Feammox(nitrite) and Feammox(nitrate) could be neglected. The removal efficiency of ammonium reached 52.3% in the anammox-derived Feammox bioreactor. Feammox played the main role in the removal of ammonium, while PN/A and nitrification played a secondary role due to the leakage of oxygen. The cycle of Fe(III)/Fe(II) was triggered between Feammox and the chemical oxidation of Fe(II) due to the low oxygen in the bioreactor. This resulted in low demand for Fe(III) dosage, only 5.8% of the theoretical demand in Feammox.

## Data Availability Statement

The datasets presented in this study can be found in online repositories. The names of the repository/repositories and accession number(s) can be found at: https://www.ncbi.nlm.nih.gov; PRJNA839877.

## Author Contributions

LH: investigation and writing-original draft. XC: investigation. GQ: conceptualization. MZ: conceptualization and writing-review and editing. YD and JL: writing-review and editing. KX: conceptualization, funding acquisition, project administration, methodology, writing-original draft, review, and editing. All authors contributed to the article and approved the submitted version.

## Funding

This study was supported by the Open Research Fund Program of the Key Laboratory of Cleaner Production and Integrated Resource Utilization of China National Light Industry (No. CP2021YB03).

## Conflict of Interest

The authors declare that the research was conducted in the absence of any commercial or financial relationships that could be construed as a potential conflict of interest.

## Publisher's Note

All claims expressed in this article are solely those of the authors and do not necessarily represent those of their affiliated organizations, or those of the publisher, the editors and the reviewers. Any product that may be evaluated in this article, or claim that may be made by its manufacturer, is not guaranteed or endorsed by the publisher.
