# Supplementary material for: Achieving Ammonium Removal Through Anammox-Derived Feammox With Low Demand of Fe(III)
Source: Front Microbiol. 2022 Jun 27;13:918634. doi: 10.3389/fmicb.2022.918634 (PMC9271925; doi:10.3389/fmicb.2022.918634)
Supplement: Supplementary file 1 [file Image_1.PDF]

Supplementary Material

1 Supplementary Figures

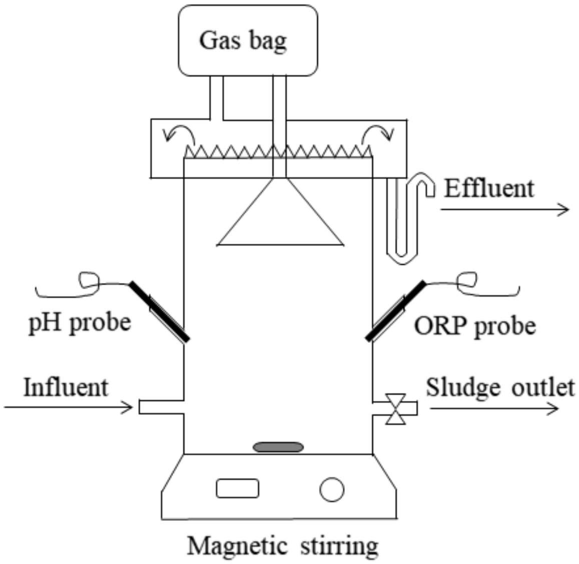

Supplementary Figure 1. Up-flow bioreactor with a three-phase separator.

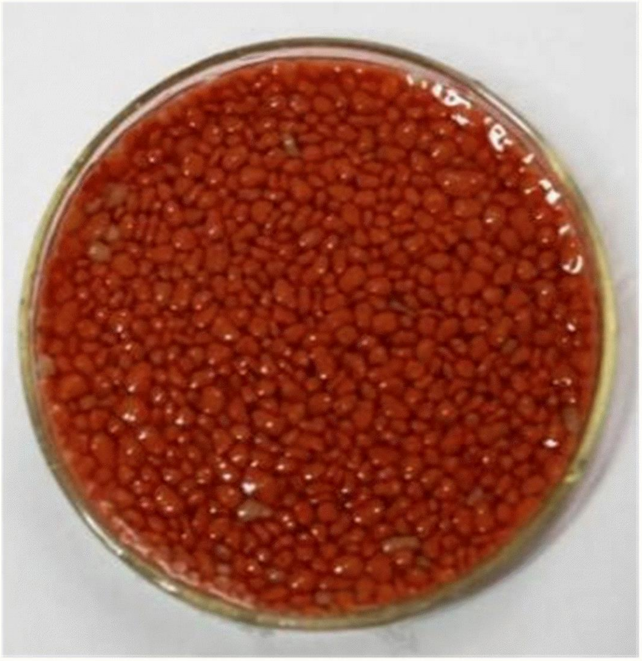

Supplementary Figure 2. Granular anammox sludge used in this study. The inoculated sludge was taken from a pilot plant of sewage treatment plant. Most of the granules were about 2 mm in diameter.

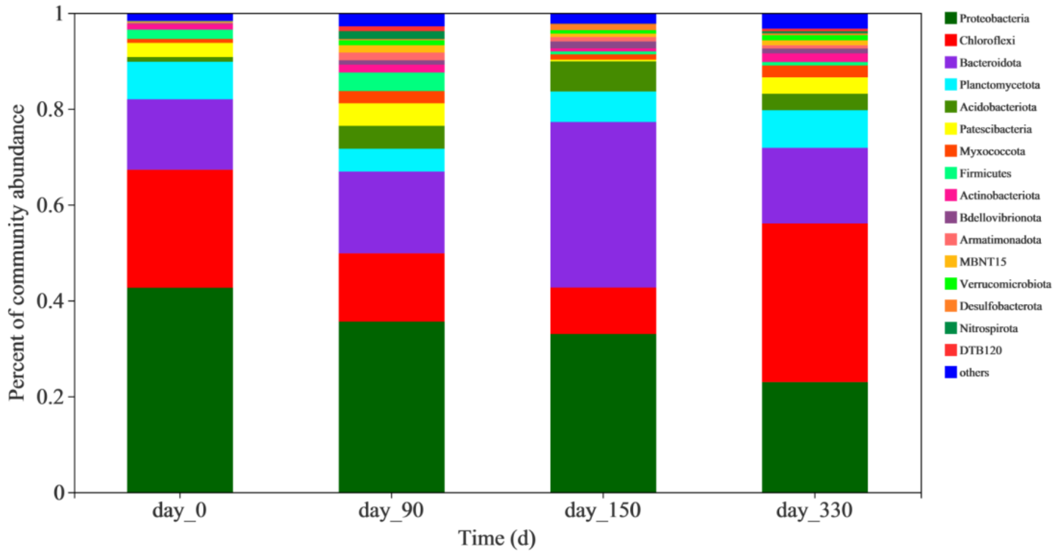

Supplementary Figure 3. Microbial community of the sludge in the Feammox bioreactor and their abundances at Phylum level.
